# Ways to Drunkenness

**Published:** 1860-01

**Authors:** 


### HALL'S JOURNAL OF HEALTH.

**Our Legitimate Scope is almost boundless: for whatever begets pleasurable and harmless feelings, promotes Health; and whatever induces disagreeable sensations, engenders Disease.**

WE AIM TO SHOW HOW DISEASE MAY BE AVOIDED, AND THAT IT IS BEST, WHEN SICKNESS COMES, TO TAKE NO MEDICINE WITHOUT CONSULTING A PHYSICIAN.

Vol. VII.] JANUARY, 1860. [No. 1.

### WAYS TO DRUNKENNESS.

A BEAUTIFUL Knickerbocker custom is it for a gentleman on New Year's day to call on his lady acquaintances as a token of respectful remembrance, intimating thereby that he desires this acquaintance to be continued, and he judges from the manner of his reception whether such a continuance would be agreeable or not. Some ladies vouchsafe the pleasurable certainty by returning the call, the next day, to those whom they specially desire to remain their recognized friends.

With this commendable custom has grown up a usage of questionable expediency, that of having a table spread with various delicacies—wines, cordials, and brandies being considered by some as indispensables. The result being, that in the joyousness of the interview, not lasting, generally, over two or three minutes, a sip is taken of this, that, and the other, and being repeated at every dwelling, gentlemen, ere they are aware of it, find themselves unmistakably drunk, and the Rubicon once crossed, the ice once broken, the *morale* once lost, life ends in the gutter.

Seeing these objections, some thoughtful persons have for years removed the wine-cup, and replaced it with coffee, lemonade, or pure cold water, the eatables remaining the same. Let every mother, who has a son who might be misled, be equally considerate; and if she have not a son herself, let her remember that some other sister-woman has a son to be lost or saved, and act accordingly.

Aside from this objection, the custom is a beautiful one, beautiful morally, beautiful socially, especially in large cities, where the press of duties and the rush of business insensibly defer intended calls on prized friends until weeks and months have passed away, when the shame of the delinquency comes in, excuses are framed, and finally it is concluded the interval has been so long that the acquaintance may as well be dropped, and the parties meet indifferently ever after. But when a day in a year is fixed by common consent for "adjusting these arrearages," for making out a list of pleasant faces whose remembrance it is not wished should pass away, the very work of casting about for the names of the prized has a sweetness about it which of itself is worth much.

But when a lady lays her head on her pillow on New Year's night, the gladness of the day is very liable to be followed with recollections which are painfully sad. Some faces she expected to see did not present themselves; a year before, how merry they were, how joyous was the greeting! But one has removed to a distant part of the country; to another, reverses have come, pecuniary or social; a third has gone upon the returnless journey, while here and there one is found who has chosen to drop the acquaintance without any assignable reason.

Then there are maiden ladies, who, some years ago, numbered their callers by dozens and scores, and even hundreds; but for a few years past they have fallen off in geometrical progression, and now the diminution is really frightful. Formerly, when youth and beauty were theirs, the door-bell began to tingle as soon as the clock struck nine of the morning, with scarcely an intermission until it verged toward midnight. But now how great the change! Merry voices are heard outside, but they do not greet their ears; brisk footfalls sound on the pavement, but they do not stop at their doors, and a weary forenoon has almost passed away with only one or two visitors to break the disturbing monotony, and former visions begin to assume more tangible shapes, and the embodied idea stands out in high relief—*Passée!*

But yonder comes a poor unfortunate bachelor; his hat is faultlessly sleek, as faultlessly shine his boots. Christadora has supplied him with one of his most natural wigs, and to the whiskers Phalon has imparted the deepest, glossiest black. Allen has given him teeth, whose perfection of finish vies with dame Nature herself; in fact, at a short distance the man is without a fault; but, on a nearer view, it is seen that youth has fled from the face ;. the eye is no more joyous; the nimble step, the supple joint, the rollicking air, all are gone, and as for the poor heart, why, there is nothing in it! it is as hollow as his head; for in the heyday of youth, when he had his pick and choice of an hundred, he was soft enough to imagine that he was entitled to a piece of perfection; and while he was looking around for it, this one, whom he thought almost so, was caught up by a wiser man; then the second best, and the third best, and so on, until the remnant were so common, in his judgment, that he went off on other explorations, where the same fatality followed him, and now he has come back to the old stamping-ground. confident that he will receive the greetings as of yore. But he has got old in the mean time, changes have come, new names are on the doors, and if now and then the name is the same, the once merry occupant has mated with another, and anon his face becomes a mile long; the corners of his lips are turned downward; in his meditations he has forgotten the day and the occasion; he walks along, a veritable "abstraction," and, when too late, soliloquizes in reality:

> "I feel like one who treads alone
> Some banquet-hall deserted;
> Whose lights are fled, whose garlands dead,
> And all but me departed."

Let it be then the wisdom of the reader, whether man or woman, if as yet unmated, to resolve that the first day of January, eighteen hundred and sixty, shall be the last New Year's which shall find them out of the bonds of steadying and happifying wedlock; for out of it there is no pure enjoyment, while in it there is bliss or—otherwise! according to the circumstances of the case and the wisdom of the parties. But, inasmuch as out of wedlock there is no rest and much that is calculated to wilt and wither the finer feelings of our nature, while in married life there is for the most part, in the aggregate, a world of enjoyment in cherishing the highest and most refining qualities of the human heart, it is wise to wed.

